# Supplementary material for: Enhanced North Pacific subtropical gyre circulation during the late Holocene
Source: Nat Commun. 2021 Oct 12;12:5957. doi: 10.1038/s41467-021-26218-7 (PMC8511172; doi:10.1038/s41467-021-26218-7)
Supplement: Supplementary file 1 — Supplementary Information [file 41467_2021_26218_MOESM1_ESM.pdf]

Supplementary Information for

Enhanced North Pacific Subtropical Gyre circulation during the late Holocene

by Zhang *et al.*

This PDF file includes:

Supplementary Figures 1 to 7

Supplementary Table 1

Supplementary References

Supplementary Figures

Supplementary Figure 1. (a). Independent $U^{K'}_{37}$-SST records (as listed in Supplementary Table 1) and (b). *Globigerinoides ruber* Mg/Ca SST estimate from the Okinawa Trough (colorful triangles denote the revised ^{14}C age control of each individual paleorecords correspondingly, fully provided in Source Data). (c). Comparison between the probabilistic stack (and one standard deviation error) of existing SST paleorecords in the Okinawa Trough.

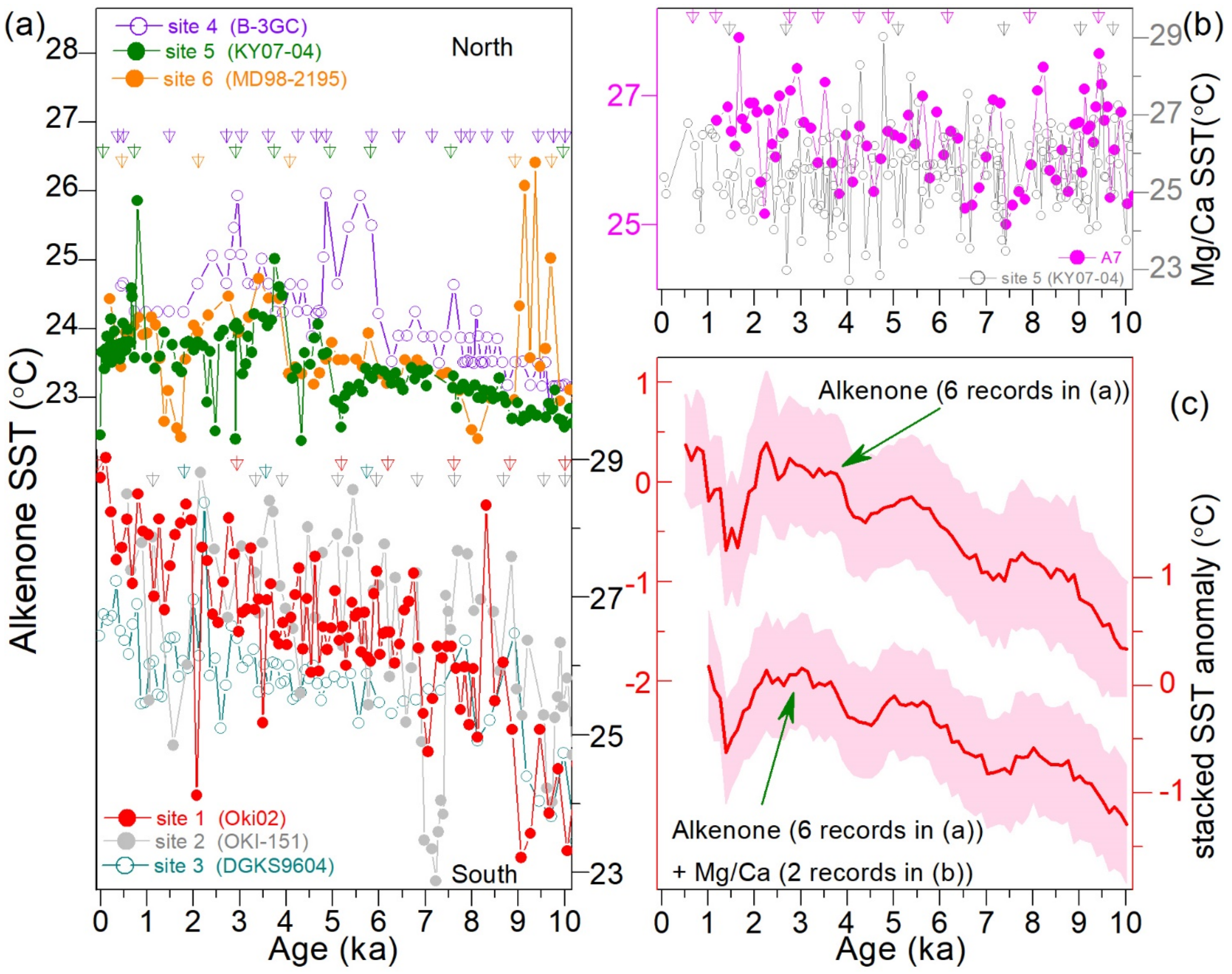

Supplementary Figure 2. (a) and (b). Compilation of published SST reconstructions (and revised ^{14}C ages, colorful triangles) in the western tropical Pacific, including both $U^{K'}_{37}$ (e.g., SST records at site 16 (ODP 1202) and site 18 (MD06-3075) and foraminifera *Globigerinoides ruber* Mg/Ca SST records (Supplementary Table 1). Note that those shown in (a) on the left panel were used to generate the probabilistic stack as given in Fig. 2c, and others on the right panel and those in (b) (see details in Supplementary Table 1) are also marked by the black cycles in Fig. 1a for further reference. (c). Comparison between the probabilistic stack (with one standard deviation error) of existing SST records over the Western Pacific Warm Pool (WPWP) by using 6 records on the left side in (a)) and western tropical Pacific (19 records in total, including 6 records on the left side in (a) and all other 13 records in (b)).

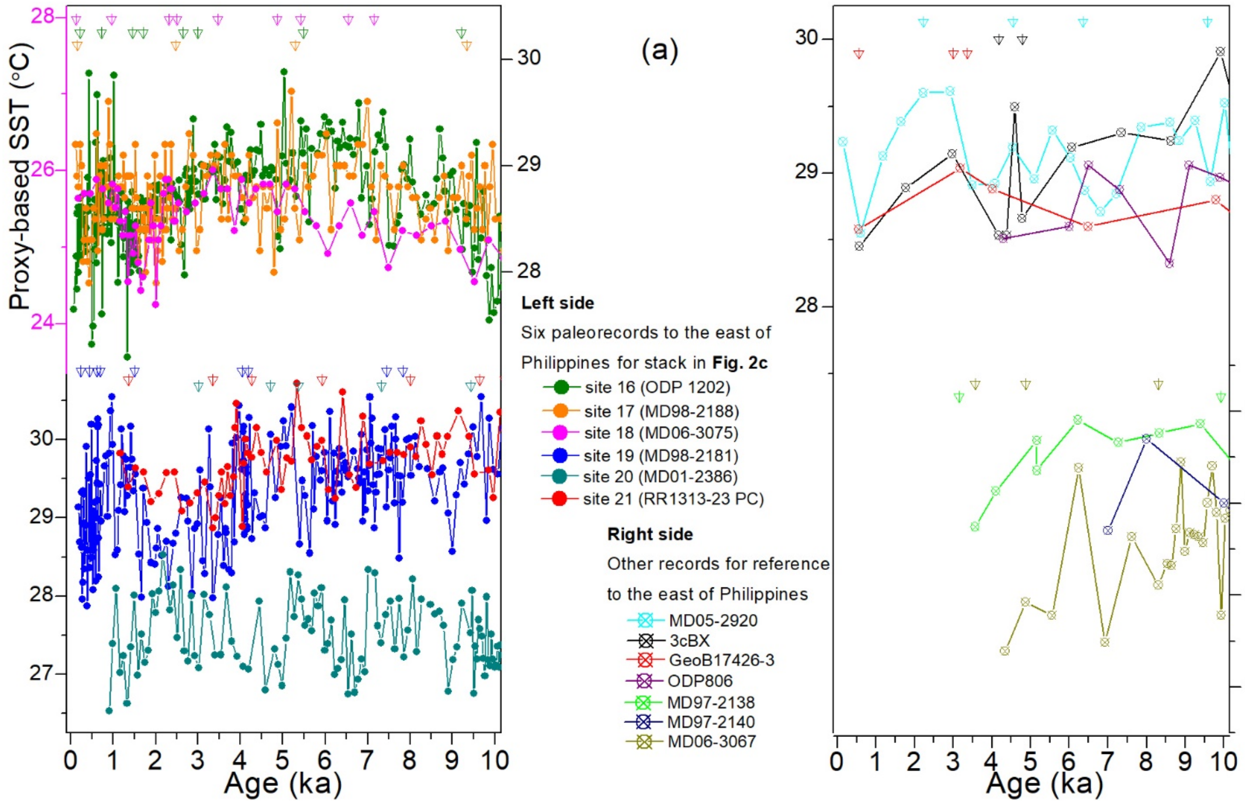

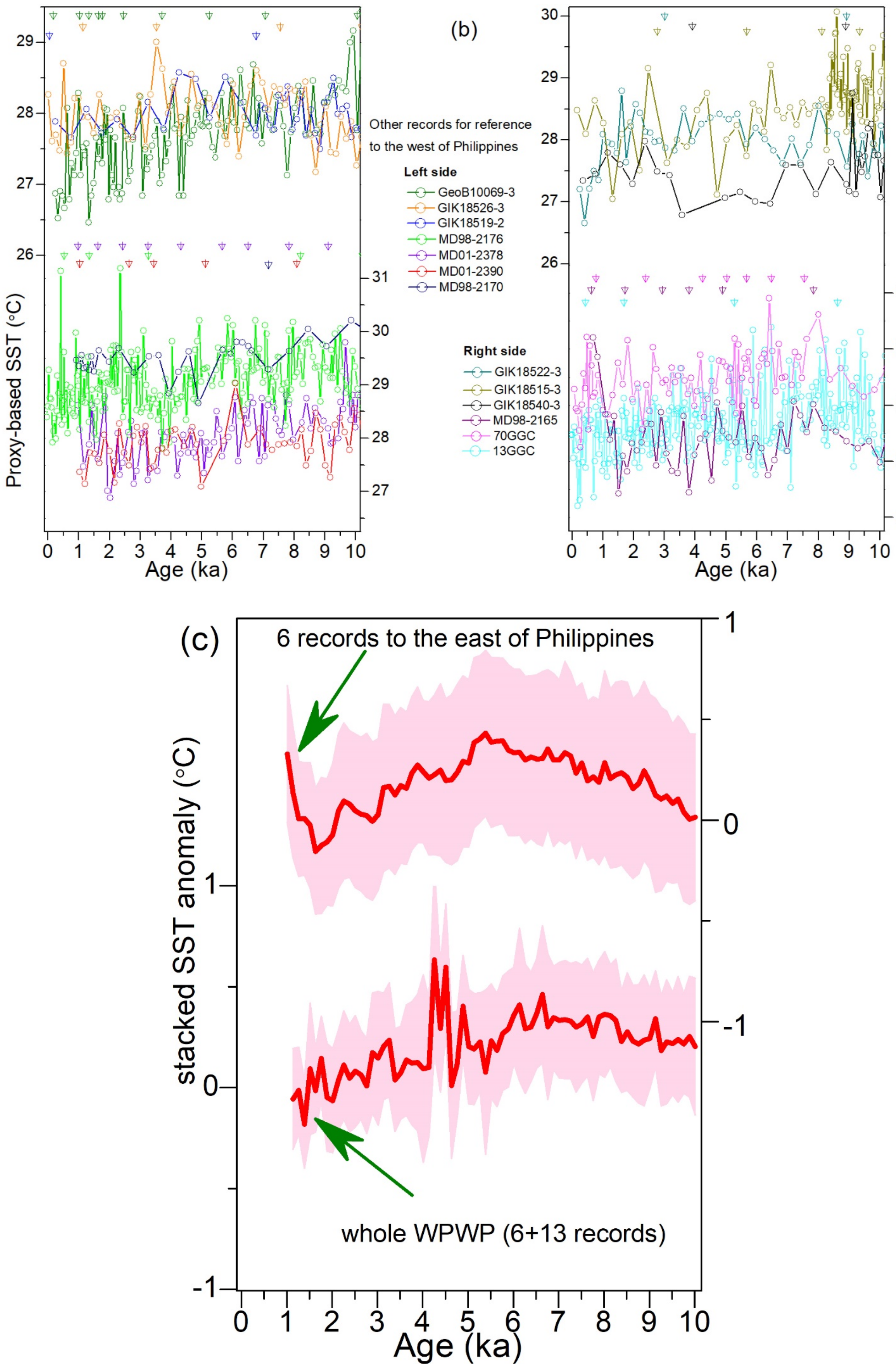

(b)
Proxy-based SST (°C)
Age (ka)
Other records for reference
to the west of Philippines
Left side
GeoB10069-3
GIK18526-3
GIK18519-2
MD98-2176
MD01-2378
MD01-2390
MD98-2170
Right side
GIK18522-3
GIK18515-3
GIK18540-3
MD98-2165
70GGC
13GGC
(c)
6 records to the east of Philippines
whole WPWP (6+13 records)
stacked SST anomaly (°C)
Age (ka)

Supplementary Figure 3. Comparison between proxy-converted sea surface temperature (SST) values for the uppermost samples (scattered dots) of compiled cores down the Okinawa Trough and long-term (1955–2012 AD) monthly averaged SST at the nearby sites (connected cycles) from the World Ocean Atlas 2013 dataset[1]. Dashed lines represent the annual mean SST values at these observational sites correspondingly.

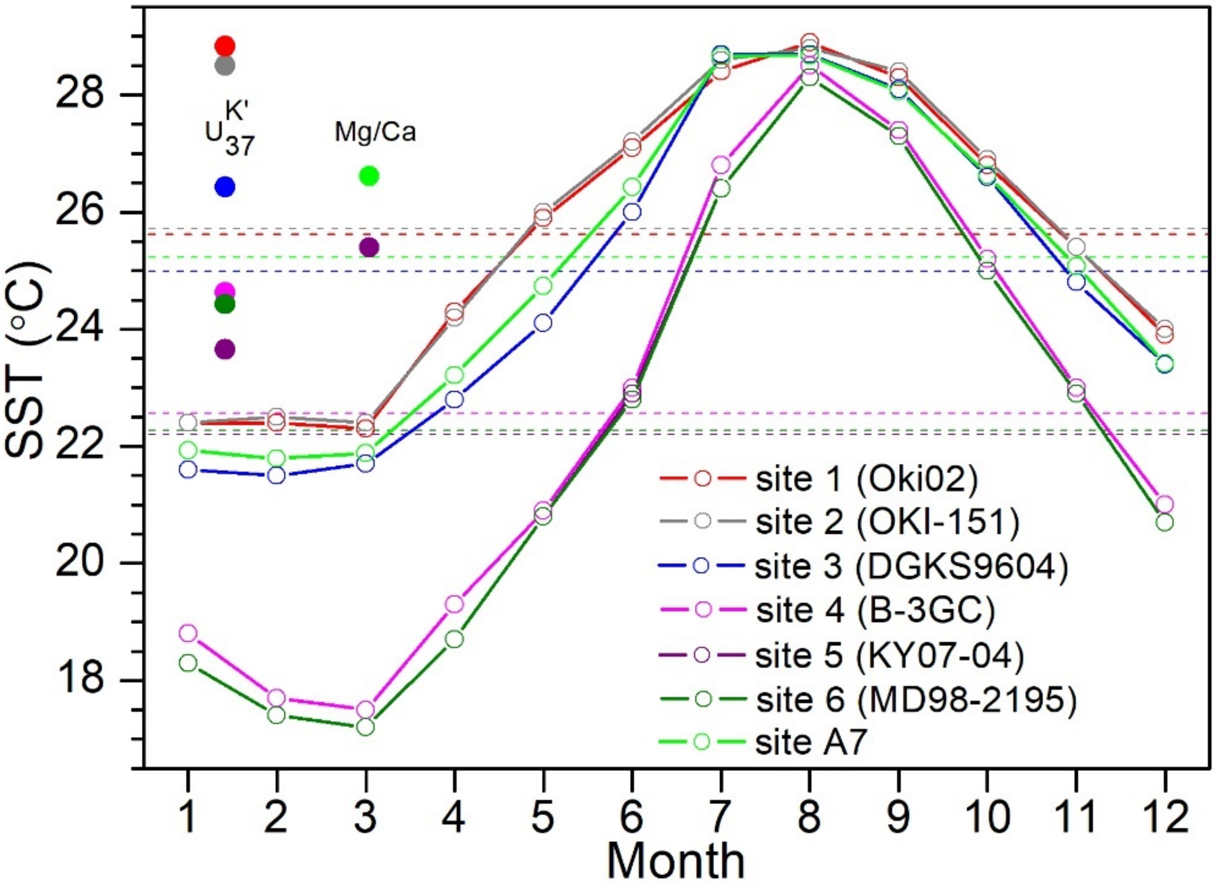

Supplementary Figure 4. Sketch map shows sea surface temperature (SST) anomalies and the position of atmospheric pressure systems (such as the Aleutian Low, abbreviated as AL, and North Pacific Jet, by following the conceptual model[2]) over the North Pacific in (a) El Niño years and (b) La Niña years (both using the five strongest events since 1951 AD defined by existing dataset at https://www.ncdc.noaa.gov/teleconnections/enso/indicators/soi/). Red stars mark three core sites over the northeast Pacific margin (as in Fig.1a of the main text) for reference, black dots and polygon present paleoclimate records in western North America that experienced wet conditions after 4–5 ka.

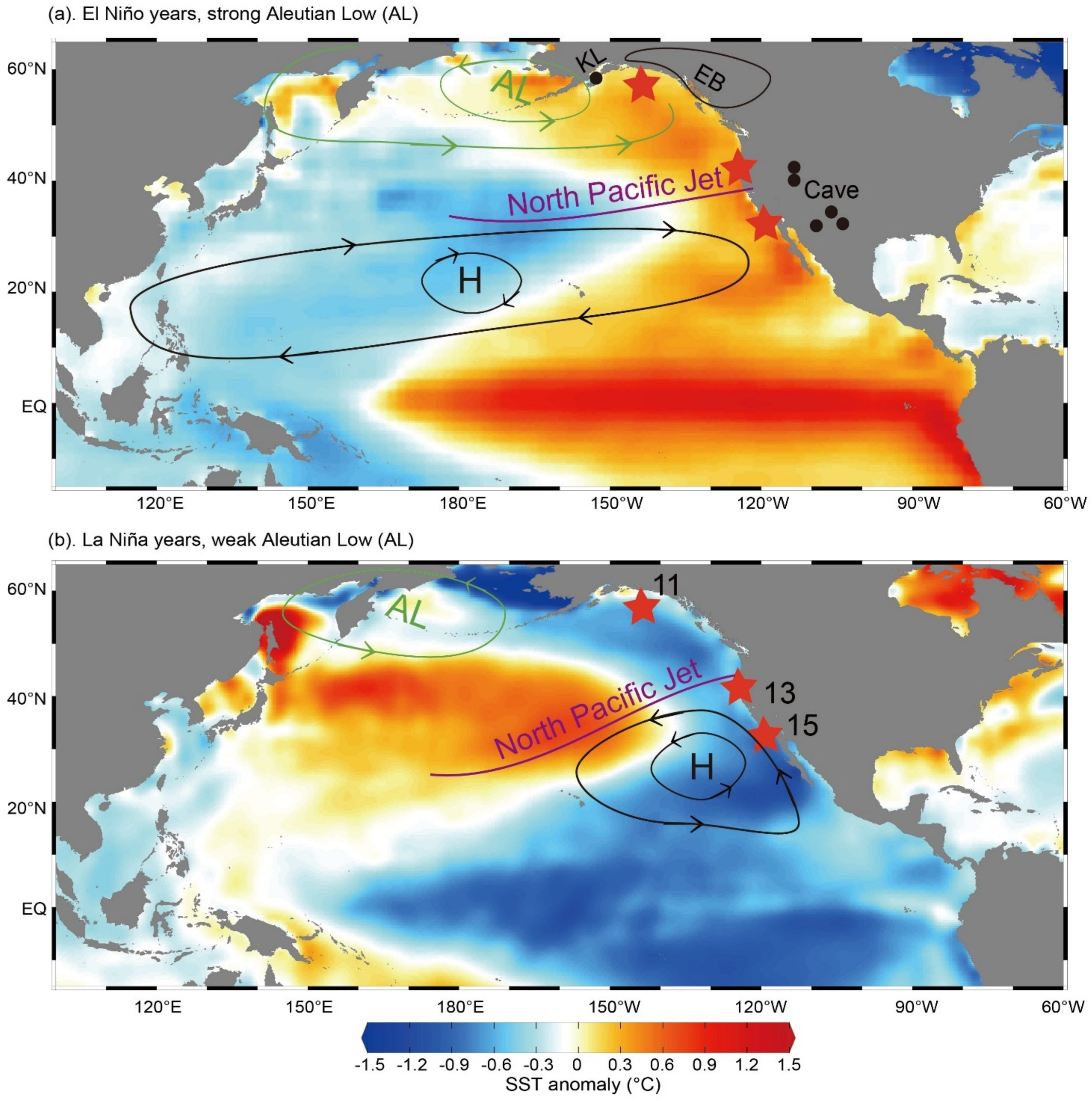

Supplementary Figure 5. Holocene change of eustatic sea level (ESL) in (a) the northeast (NE) and northwest (NW) Pacific. Grey bar highlights the significant drop of sea level prior to ~7 ka (note the error bars present uncertainty in both age control (horizontal axis) and sea level (vertical axis)).

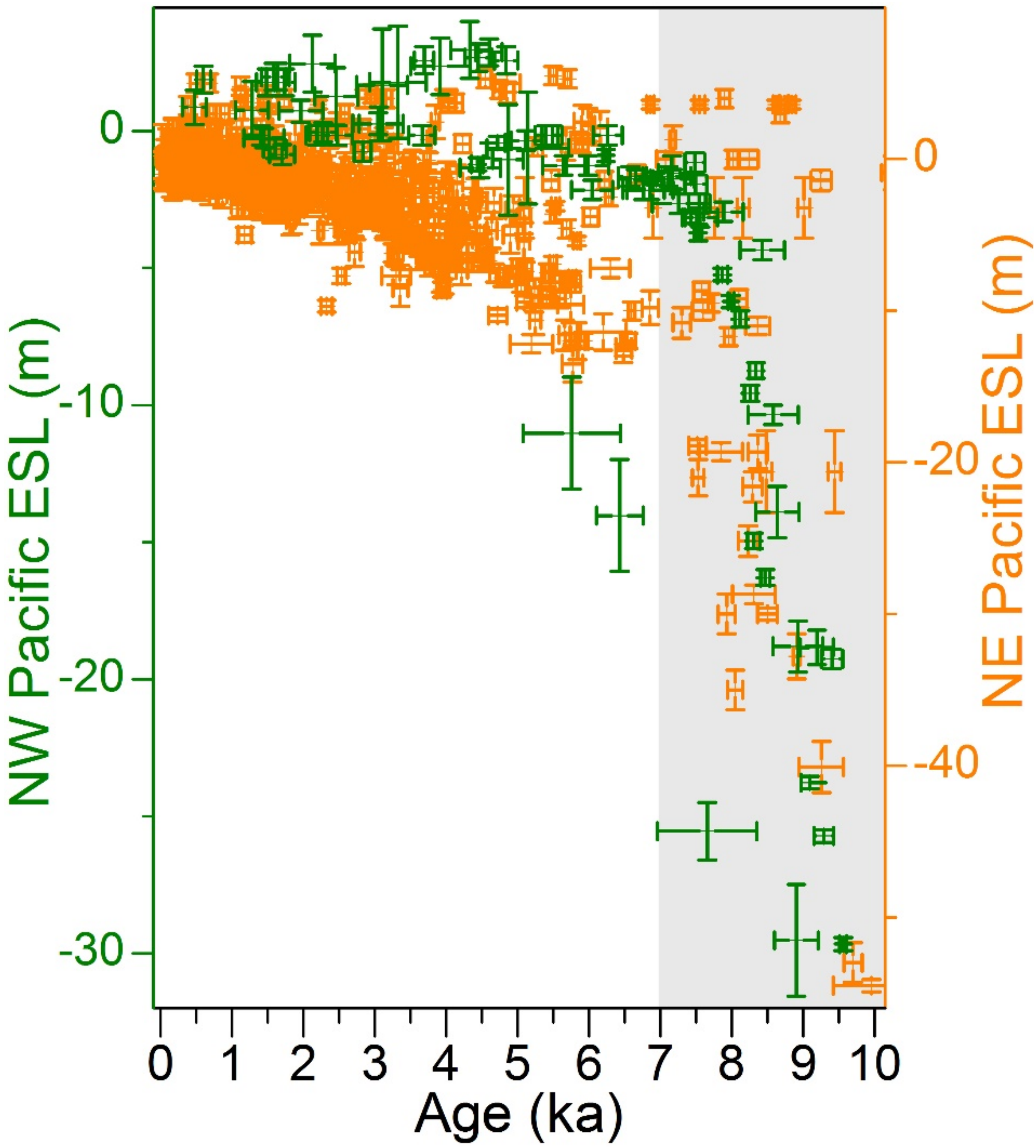

Supplementary Figure 6. Comparison between stacked SST anomaly in the Kuroshio Current (KC) path (a) and its difference with stacked SST in the Western Pacific Warm Pool (c), as well as Cariaco Titanium (Ti) record (b, binning-averaged by using a 125-year-window). Lower panel, correlation between (left) stacked SST anomaly in the KC path and Ti record, (right) meridional SST gradient and Ti record within the time window of past 7 ka (outlined by the grey box in the upper panel).

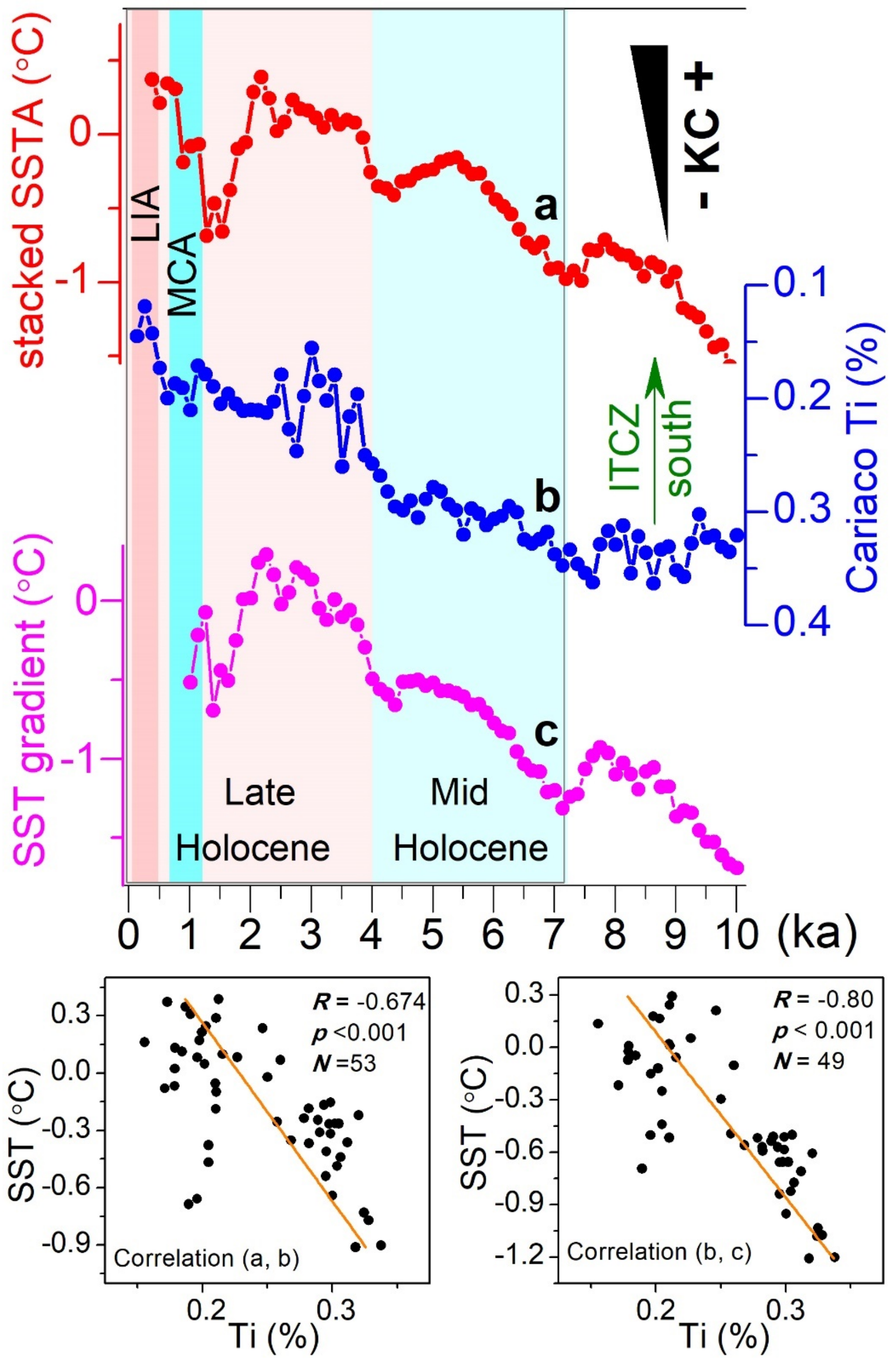

Supplementary Figure 7. Comparison of $U^{K'}_{37}$ (upper panel, site 3)[3–5], foraminifera *Globigerinoides ruber* Mg/Ca (middle panel, site 5) SST records by different equations[6, 7] (left) and their calculated anomalies (right), as well as outputs of probabilistic stack accordingly (lower panel).

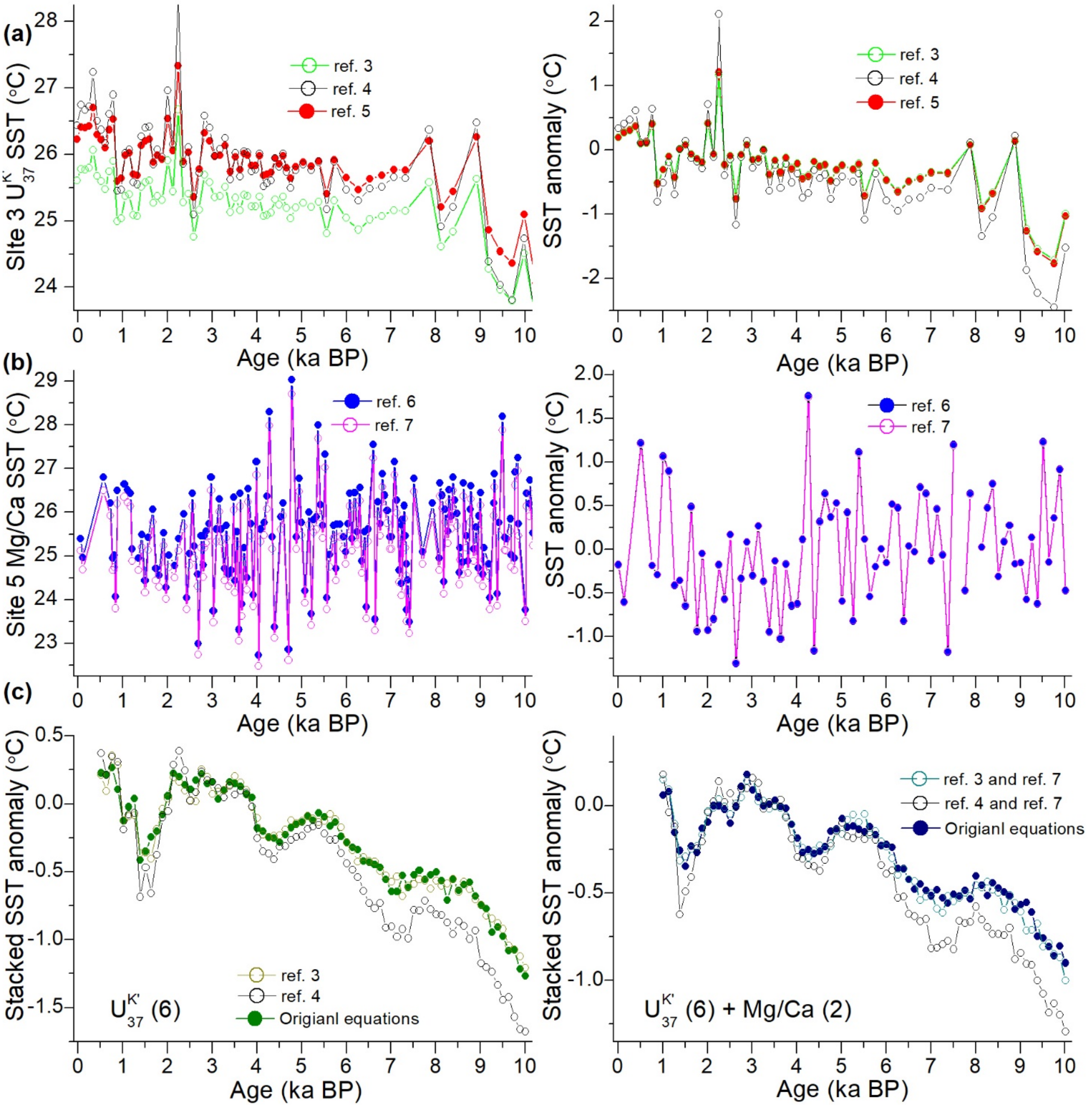

Supplementary Table 1. Summary of all complied SST records (original references of each paleorecords are listed in Source Data correspondingly).

Site No.	Sediment core	Lat. (°N)	Long. (°E)	Water depth (m)	Numbers of ^{14}C age points within ~10 ka	Resolution (in years)	Proxy
Central and northern Okinawa Trough (Kuroshio Current) (note only sites 1-6 are used to compute probabilistic stack as shown in Figs.2b and 4c)							
1	Oki02	26.07	125.2	1612	7	~100	$U^{K'}_{37}$
2	OKI-151	26.11	125.52	2013	11	~150	$U^{K'}_{37}$
3	DGKS9604	28.28	127.02	766	3	~145	$U^{K'}_{37}$
4	B-3GC	31.48	128.52	555	20	~145	$U^{K'}_{37}$
5	KY07-04 PC-01	31.64	128.94	758	8	~80	$U^{K'}_{37}$
6	MD98-2195	31.64	128.94	746	5	~165	$U^{K'}_{37}$
	KY07-04 PC-01	31.64	128.94	758	8	~60	Mg/Ca
	A7	27.82	126.98	1264	6	~130	Mg/Ca
7	38002	37.99	122.50	49	5	~20	$U^{K'}_{37}$
Kuroshio-Oyashio Interfrontal Zone (as individually shown in Fig.4b)							
8	C0011	32.83	136.88	4050	Biostratigraphy	400	$U^{K'}_{37}$
9	KR02-06 St. A GC	36.03	141.78	2224	11	~60	$U^{K'}_{37}$
10	Station 5	40	165.07	5498	4	700	$U^{K'}_{37}$
Northeastern Pacific (California Current and Alaska Current) (as shown in Fig.3)							
11	EW0408-85JC	59.56	-144.15	682	14	~300	$U^{K'}_{37}$
12	JT96-06 PC	48.91	-126.89	920	2	~500	$U^{K'}_{37}$
13	ODP1019	41.68	-124.93	989	6	~140	$U^{K'}_{37}$
14	ODP1017	34.53	-121.11	955	2	~350	$U^{K'}_{37}$
15	ODP 893	34.29	-120.04	576	19	~800	$U^{K'}_{37}$

Western Pacific Warm Pool (WPWP)/KC upstream (note only sites 16-21 are used to compute probabilistic stack as shown in Fig.2c)							
16	ODP1202	24.8	122.5	1274	8	~40	$U_{37}^{K'}$
17	MD98-2188	14.82	123.49	730	5	65	G. ruber Mg/Ca
18	MD06-3075	6.48	125.83	1878	10	150	$U_{37}^{K'}$
19	MD98-2181	6.3	125.8	2114	10	50	*G. ruber* Mg/Ca
20	MD01-2386	1.13	129.79	2816	6	100	*G. ruber* Mg/Ca
21	RR1313-23PC	-4.49	145.67	712	8	150	*G. ruber* Mg/Ca
	MD05-2920	-2.81	144.50	1843	4	475	*G. ruber* Mg/Ca
	GIK18540-3	-6.87	119.58	1189	3	350	*G. ruber* Mg/Ca
	3cBX	8.02	139.64	2829	2	850	*G. ruber* Mg/Ca
	MD97-2138	1.25	146.14	1960	2	850	*G. ruber* Mg/Ca
	MD06-3067	6.53	126.50	1575	3	450	*G. ruber* Mg/Ca
	GeoB17426-3	-2.19	150.86	1368	4	2000	*G. ruber* Mg/Ca
	ODP806	0.32	159.36	2520		1200	*G. ruber* Mg/Ca
	MD97-2140	2.03	141.46	2547		3000	*G. ruber* Mg/Ca
	GIK18519-2	-0.57	118.11	1658	3	275	*G. ruber* Mg/Ca
	MD98-2170	-10.59	125.39	832	2	350	*G. ruber* Mg/Ca
	MD98-2165	-9.7	118.3	2100	9	220	*G. ruber* Mg/Ca
	GIK18515-3	-3.63	119.36	688	6	125	*G. ruber* Mg/Ca
	MD01-2390	6.6	113.4	1545	3	200	*G. ruber* Mg/Ca
	GIK18522-3	1.4	119.08	975	3	220	*G. ruber* Mg/Ca
	GeoB10069-3	-9.59	120.92	1250	10	85	*G. ruber* Mg/Ca
	GIK18526-3	-3.61	118.17	1524	5	150	*G. ruber* Mg/Ca
	BJ8 70GGC	-3.6	119.4	482	8	120	*G. ruber* Mg/Ca
	MD01-2378	-13.1	121.8	1783	10	130	*G. ruber* Mg/Ca
	MD98-2176	-5	133.4	2382	5	50	*G. ruber* Mg/Ca
	BJ8 13GGC	-7.4	115.2	594	5	45	*G. ruber* Mg/Ca

Supplementary References

1. Locarnini, R. *et al.* World Ocean Atlas 2013, Volume 1: Temperature. In NOAA Atlas NESDIS 73 (eds Levitus, S. & Mishonov, A.) (National Oceanographical Data Center, 2013).
2. Du, X. *et al.* High-resolution interannual precipitation reconstruction of Southern California: Implications for Holocene ENSO evolution. *Earth Planet. Sci. Lett.* 554, (2020).
3. Prahl, F., *et al.* Further evaluation of long-chain alkenones as indicators of paleoceanographic conditions. *Geochim. Cosmochim. Acta* 52, 2303–2310 (1988).
4. Tierney, J., Tingley, M. BAYSPLINE: A new calibration for the alkenone paleothermometer. *Paleoceanogr Paleoclimatol.* 33, 281–301 (2018).
5. Müller, P., *et al.* Calibration of the alkenone paleotemperature index $U_{37}^{K'}$ based on core-tops from the eastern South Atlantic and the global ocean (60N–60°S). *Geochimica et Cosmochimica Acta*, 62(10), 1757– 1772 (1998).
6. Hastings, D., *et al.* A comparison of three independent paleotemperature estimates from a high resolution record of deglacial SST records in the tropical South China Sea, *Eos Trans. AGU*, 82(47), Fall Meet. Suppl., Abstract PP12B-10 (2001).
7. Anand, P., Elderfield, H., & Conte, M. Calibration of Mg/Ca thermometry in planktonic foraminifera from a sediment trap time series. *Paleoceanography* 18 (2003).
